# Intracellular immunity to HIV-1: newly defined retroviral battles inside infected cells

**DOI:** 10.1186/1742-4690-2-25

**Published:** 2005-04-13

**Authors:** Yong-Hui Zheng, B Matija Peterlin

**Affiliations:** 1 Departments of Medicine, Microbiology and Immunology, Rosalind Russell Arthritis Research Center, University of California, San Francisco, San Francisco, CA, 94143-0703, USA

## Abstract

Studies of the human immunodeficiency virus type 1 (HIV-1) continue to enrich eukaryotic biology and immunology. Recent advances have defined factors that function after viral entry and prevent the replication of proviruses in the infected cell. Some of these attack directly viral structures whereas others edit viral genetic material during reverse transcription. Together, they provide strong and immediate intracellular immunity against incoming pathogens. These processes also offer a tantalizing glimpse at basic cellular mechanisms that might restrict the movement of mobile genetic elements and protect the genome.

## Background

Although it is highly pathogenic in humans, HIV-1 cannot replicate in most other species [1]. This tropism is determined primarily by whether host cells express the required cofactors. For example, by lacking a functional receptor and appropriate transcriptional machinery, mouse cells do not support infection by HIV-1. Thus, the organism resists the pathogen via a cell-based incompatibility. However, a pathogen can also be restricted by the presence of dominant inhibitory factors. They attack the incoming virus directly and block its integration into the host genome. This situation also pertains to HIV-1 in mouse cells and represents true "intracellular immunity." Importantly, this host response is more rapid than either traditional innate or adaptive immunity and can prevent the establishment of the infection.

Recent advances in our understanding of intracellular immunity have identified two different proteins, the tripartite motif protein 5α (TRIM5α) [2] and the apolipoprotein B mRNA-editing enzyme catalytic-polypeptides 3B, 3F and 3G (APOBEC3B, APOBEC3F and APOBEC3G or A3B, A3F and A3G), which collectively inactivate several retroviruses including HIV-1, simian immunodeficiency virus (SIV), hepatitis B virus and some mouse mobile genetic elements [3-7]. This review highlights these recent developments and mentions briefly additional potential blocks to retroviral replication.

## Interference and Restriction

**Figure 1:**
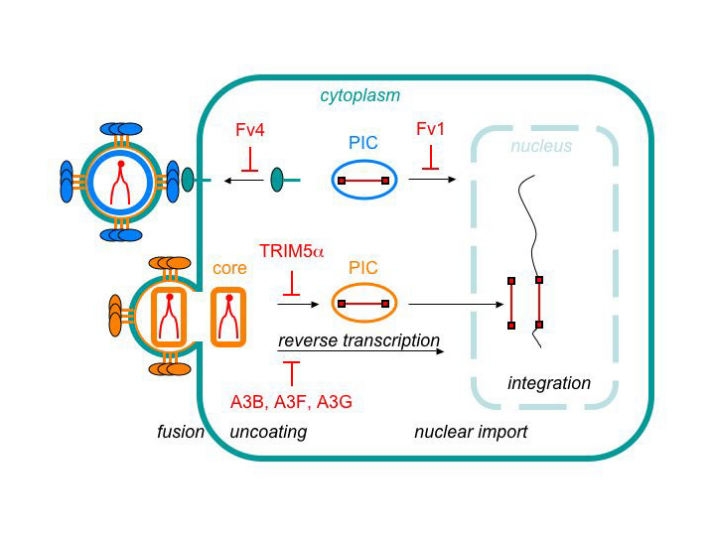
Effective intracellular immunity targets incoming viruses. Whereas the ecotropic murine leukemia virus (MLV) (represented as a viral particle in blue) encounters Fv4 and Fv1 blocks, other retroviruses such as HIV-1, SIV and EIAV (represented as a viral particle in brown) encounter TRIM5α and cytidine deaminase blocks. Fv4 prevents the entry by ecotropic MLV by sequestering the viral receptor from the cell surface. Fv1 targets MLV CA and stops the nuclear import of the viral preintegration complex (PIC). TRIM5α also targets retroviral CA and blocks uncoating. hA3B, hA3F and hA3G deaminate cytidines on newly synthesized retroviral cDNA and disrupt viral replication. Capital red letters highlight the points of inhibition. Viral structural components, nucleic acids, RNA and DNA, and intracellular events are represented in different colors.

Let us begin with some definitions and historical perspectives. Viral "interference" refers to the situation when cells, which are chronically infected with one virus or contain endogenous retroviruses, resist superinfection by other viruses bearing envelopes with a similar target specificity. This block usually results from the loss of the appropriate receptor on the cell surface. A good example of this interference is the Friend virus susceptibility factor 4 (Fv4), also known as Akvr-1, which controls the susceptibility of mice to infection by ecotropic but not other murine leukemia viruses (MLVs) [8]. This gene is located on mouse chromosome 12 [9] within an endogenous defective provirus and encodes a complete envelope [10] that shares very high sequence similarity with those from ecotropic Cas-Br-E virus and Moloney MLV [11]. This envelope then blocks the expression of the cationic amino acid transporter, which is the receptor for these MLVs, on the cell surface (Fig. 1) [12]. Of interest, MLV can only use the murine but not the human form of this receptor for entry.

**Figure 2:**
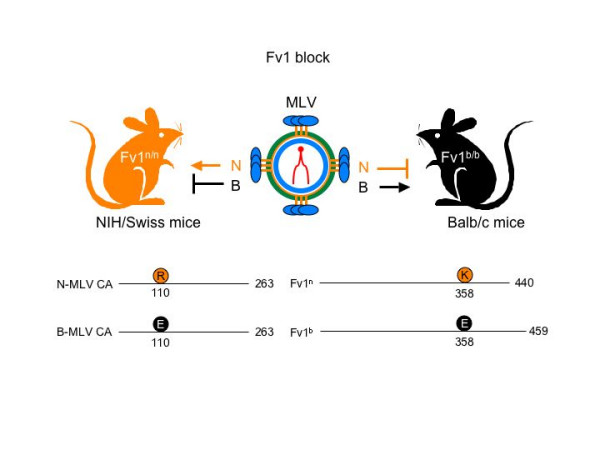
Fv1 block in MLV infection. Ecotropic MLVs (e.g. Friend MLV) fall into two categories with respect to their host range: N-tropic strains infect NIH/Swiss mice (brown) much more efficiently than BALB/c mice (black), whereas B-tropic strains display the opposite preference. Based on their susceptibility to N- or B-tropic virus, mice were classified into Fv1^n/n ^(NIH/Swiss) and Fv1^b/b ^(Balb/c) strains. These viruses differ at a single residue at position 110 in CA (presented below the NIH/Swiss mouse). These changes are matched by residues at position 358 in Fv1^n ^and Fv1^b ^proteins (presented below the Balb/c mouse).

The term "restriction" refers to intracellular blocks to viral replication. Until now, the best example has been Fv1 [13]. Like Fv4 and the less well-characterized Fv3 and Fv2, Fv1 also confers resistance of mice to the infection by MLV (Figs. 1 and 2). The Fv1 gene is located on mouse chromosome 4 [14] and encodes a protein that resembles other endogenous retroviral structural group specific antigens (Gag) (Fig. 2) [15]. Of note, during the morphogenesis and release of progeny virions, retroviral Gag polyproteins are processed by the viral protease into distinct subunits, namely matrix (MA), capsid (CA) and nucleocapsid (NC). Whereas MA and CA form the outer shell and inner core of mature viral particles, NC packages viral genomic RNA into the core [16]. After entry and uncoating in newly infected cells, many structural proteins remain associated with viral enzymes (reverse transcriptase, RT and integrase, IN) and RNA in a large (2 mDa) preintegration complex (PIC).

Alleles of Fv1 in Balb/c (Fv1^b/b^) and NIH/Swiss (Fv1^n/n^) mice result in resistance to N- and B-tropic strains of MLV, respectively, which maps to position 110 in CA (Fig. 2) [17]. A recent structural analysis revealed that this residue is located at the outer face of the core structure of CA with easy access to cellular proteins [18]. On Fv1, the key residue for this restriction was mapped to position 358 (Fig. 2) [19]. Although binding between CA and Fv1 has not been demonstrated, they could interact as higher order structures, especially since CA and Gag form oligomers, in the case of CA, hexagonal lattices of the viral core. As heterozygous Fv1^n/b ^mice block infection by both viruses, resistance is dominant [20]. Conversely, NB-tropic MLV can infect all these mice. Of interest, this restriction is saturable with high levels of CA from either virus [21], implying that amounts of Fv1 or its cofactor/s are limiting. As described below, one of these cofactors could be TRIM5α [22]. As a result of these interactions, Fv1 is thought to block the disassembly of CA and the normal movement of the PIC into the nucleus (Fig. 1) [23,24].

### TRIM5α

**Figure 3:**
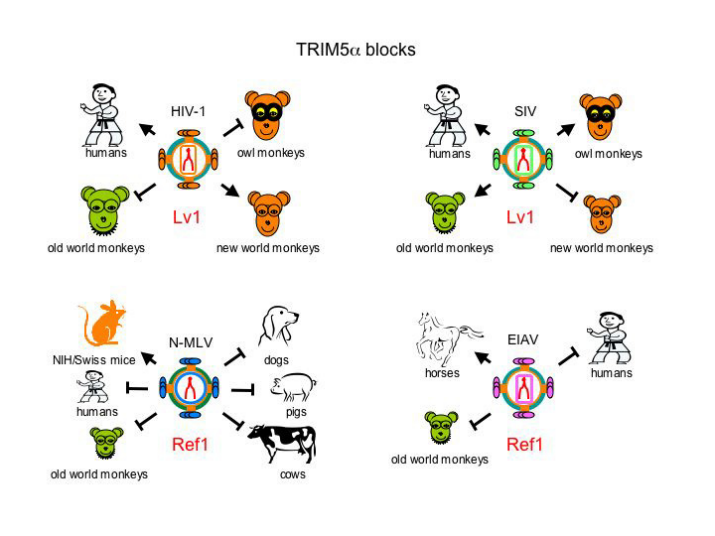
Blocks to retroviral replication by different TRIM5α proteins from several species. The replication of HIV-1 is blocked by TRIM5α from old world monkeys and owl monkeys, but not human and new world monkeys (left top panel). The replication of SIV is blocked by TRIM5α proteins from new world monkeys, but not from humans, old world monkeys, and owl monkeys (right top panel). The replication of N-MLV is prevented by TRIM5α proteins from dogs, pigs, cows, old world monkeys, and humans, but not mice (left bottom panel). The replication of EIAV is blocked by TRIM5α from human and old world monkeys, but not horses (right bottom panel). In all cases, arrows indicate no inhibition.

Fv1 is not the only genetic system conferring intracellular immunity against a retroviral infection. For example, the replication of N-tropic MLV and the equine infectious anemia virus (EIAV, a lentivirus) is also inhibited in human cells [25], as is that of the primate lentiviruses HIV-1 and SIV from rhesus macaques (SIV_mac_) in cells from different monkeys (Fig. 3) [26-29]. For example, HIV-1 does not grow in old world monkeys, which include African green monkeys and rhesus macaques, and SIV_mac _does not infect new world monkeys, which include squirrel monkeys and common marmosets (Fig. 3) [26-29].

Interestingly, these blocks resemble Fv1 restriction in several ways. First, viral replication is impaired at the step of reverse transcription [25-28]. Second, CA is also targeted. The residue at position 110 in CA also determines the restriction of N-tropic MLV in human cells [25] and that of HIV-1 in rhesus macaque cells is abrogated when its CA is replaced by that from SIVmac [29]. Third, because heterokaryons between non-restrictive and restrictive cells maintain the inhibition, this restriction is dominant [27,28]. Finally, these blocks are saturable. However, since no Fv1-related gene could be found in primate cells, blocks to N-tropic MLV and EIAV in human cells were thought to be due to the restriction factor 1 (Ref1) [25], and those to HIV-1 and SIV in monkey cells to the lentivirus susceptibility factor 1 (Lv1) (Fig. 3) [27].

Indeed, Ref1 and Lv1 share additional similarities in blocking retroviral replication. For examples, both restrictions can be attenuated by chemicals that disrupt the integrity of mitochondrial membranes [30,31], and they can be saturated by the same virus like particles (VLPs) [32]. Using a functional complementation assay, Lv1 was first identified as the rhesus macaque TRIM5α (macTRIM5α) gene [2]. Later, by eliminating TRIM5α transcripts from old world monkey and human cells with small interfering RNA (siRNA), the Lv1 and Ref1 blocks were also abrogated [33]. Further studies revealed that hTRIM5α (from humans), macTRIM5α and agmTRIM5α (from African green monkeys) restrict the replication of different viruses, which were assigned previously to Lv1 and Ref1 (Fig. 3) [22,33-35]. Thus, Ref1 and Lv1 are species-specific variants of TRIM5α.

**Figure 4:**
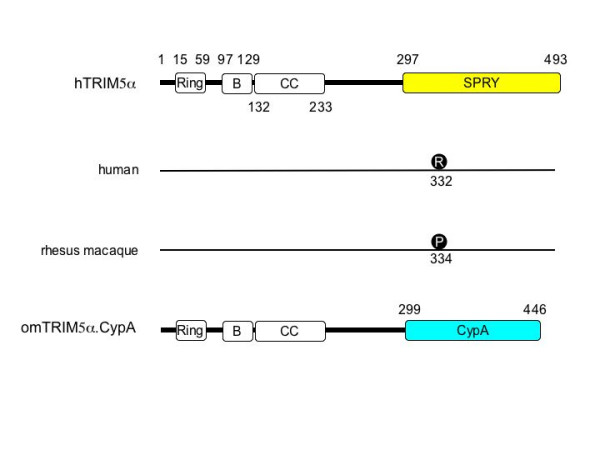
Schematic representations of hTRIM5α, macTRIM5α, and omTRIM5a. CypA proteins. hTRIM5α contains 493 residues and four conserved motifs, whose positions are given. They are the RING domain, B box, coiled-coil and SPRY domains. The latter domain is required for species-specific restriction of primate lentiviruses and is diagrammed in red. A key residue in this domain, which is the arginine at position 332 in hTRIM5α, or the proline at position 334 in macTRIM5α, is responsible for its species-specific inhibition of lentiviral replication. omTRIM5α from owl monkeys contains an N-terminal omTRIM5α sequence to position 299, linked in-frame to the entire CypA gene (147 residues)(omTRIM5α.CypA).

The hTRIM5α protein contains 493 residues (Fig. 4) and belongs to the large tripartite motif (TRIM) family that consists of 37 genes, which include the promyelocytic leukemia (PML or TRIM19) protein [36]. By alternative RNA splicing, they produce 71 different transcripts. For example, the human TRIM5 gene is expressed as hTRIM5α, β, γ, σ, ε, and ζ. Although little is known of their function, they contain three distinctive structural motifs, a RING Zn^++ ^finger, one or two B-box Zn^++ ^finger, and an α-helical coiled-coil (CC) region (Fig. 4). For this reason, they are also called the RING finger:B box:Coiled-coil (RBCC) family proteins. The RING finger motif features a cysteine-rich consensus, which contains two interleaved Zn^++^-binding sites [37]. Many RING finger proteins act as E3 ubiquitin ligases and play key roles in protein degradation. For example, Ring-box-1 (Rbx1) is an essential component of the Skp1:cullin-1:F-box (SCF) complex. Additionally, TRIM5σ displays E3 ligase activity in vitro [38]. B-boxes, which consist of one Zn^++^-binding site and a B1 or B2 motif [39], orient the CC motif that mediates protein-protein interactions. Indeed, TRIM proteins form oligomers [36]. In addition, TRIM5α contains a SPRY domain at its C-terminus (Fig. 4). The SPRY domain was originally identified in the splA kinase of *Dictyostelium *and the rabbit ryanodine receptor [40], and belongs to the subclass of the B30.2 or RFP-like domains. In butyrophilin, the B30.2 domain, which contains 170 residues, is involved in ligand binding [41]. Of interest, TRIM proteins localize to particular cellular compartments where they form discrete structures. Whereas TRIM19 assembles discrete PML oncogenic domains (PODs) in the nucleus, TRIM5α can form cytoplasmic bodies [36].

Although they share 87% sequence similarity, only macTRIM5α but not hTRIM5α blocks HIV-1 replication [2]. This species-specific restriction was mapped to the SPRY domain [42-44]. Through genetic analysis, it was revealed that this SPRY domain has experienced dramatic mutations during primate evolution [45] and contains four variable regions V1, V2, V3, and V4 [46]. The change of a single residue (R332P) in V1 abolished the inhibition of HIV-1 replication by hTRIM5α [43,44], which suggests that the SPRY domain is responsible for its targeting of CA. Although no binding between the SPRY of rhTRIM5α and HIV-1 CA has been demonstrated, the findings with TRIM5α from owl monkeys (omTRIM5α) support such direct interactions [47,48]. A rather complicated story follows. Cyclophilin A (CypA) is an eukaryotic peptidyl-prolyl cis-trans-isomerase. It binds an exposed proline-rich loop in CA of HIV-1 and is critical for its replication in human cells [49,50]. In contrast, the ability to bind CypA restricts HIV-1 replication in owl monkey cells. Owl monkeys are atypical new world monkeys because their Lv1 inhibits HIV-1 but not SIV_mac_. Although this block to HIV-1 replication is abrogated when the interaction between CA and CypA is prevented by mutations in CA or by cyclosporin A treatment in owl monkey cells, the same manipulations increase effects of Ref1 on HIV-1 in human cells [51]. The explanation for these differences came with the cloning of the omTRIM5α gene. Instead of the SPRY domain, it contains the complete CypA gene [47,48] (Fig. 4). Thus, owl monkey cells express a fusion protein between omTRIM5α and CypA (omTRIM5α.Cyp), which most likely arose from a retrotransposition of the CypA gene into the omTRIM5α locus by the long interspersed nuclear elements-1 (LINE-1 or L1). In conclusion, CA and omTRIM5α interact via this CypA domain and restrict HIV-1 replication in owl monkeys.

These studies suggest that Fv1 and TRIM5α might interact directly with CA to block incoming viruses. However, in contrast to Fv1, which blocks nuclear entry and integration of the provirus [23,24], TRIM5α inhibits viral replication at a step before reverse transcription (Fig. 1). It is puzzling why such differences exist. An answer might lie in the observation that MLV, but not HIV-1, retains its CA in the reverse transcription complex [52,53]. Thus, the uncoating of HIV-1 could proceed much faster than that of MLV. Once the core structure is destroyed, the reverse transcription complex could become more susceptible to TRIM5α. TRIM5α could then trigger the proteasomal degradation of PIC. This model also offers an explanation of the enhancement of viral replication when target cells are treated with proteasomal inhibitors [54]. Further details await studies of other proteins that interact with Fv1 and TRIM5α, their enzymatic properties and trafficking in cells.

## Cytidine deaminases

In addition to Fv1 and TRIM5α, host cells have developed additional mechanisms to protect themselves from viral invasion. The next important block involves nucleic acid editing of viral reverse transcripts. For a long time, it had been noted that retroviruses contain a high frequency of G to A transitions [55,56]. In certain strains of HIV-1, up to 60% of all guanidines are replaced by adenines [57]. Previously, this G to A hypermutation was attributed to the high error rate of reverse transcriptase and the imbalance in dCTP pools in cells [58]. However, we now know that host cellular cytidine deaminases are responsible.

In parallel, mutant HIV-1 lacking the viral infectivity factor (Vif) (HIV-1ΔVif) can only replicate in certain T cell lines, which are called "permissive" cells. In other "non-permissive" cells, only wild type HIV-1 but not HIV-1ΔVif can replicate [59,60]. Because heterokaryons between permissive and non-permissive cells do not support the replication of HIV-1ΔVif, there exists a dominant inhibitor in these non-permissive cells [61,62]. By subtractive cloning between non-permissive CEM and permissive CEM-SS T cells, the inhibitory factor was identified as the human A3G (hA3G) protein [3]. Later, its close relatives hA3F, and to a lesser degree, hA3B, were found to possess similar anti-viral activities [4-7].

The human APOBEC family comprises 10 proteins, among which are the founding member APOBEC1 (A1) and the activation induced deaminase (AID) [63]. They contain one (e.g. APOBEC1 and AID) or two (e.g. hA3F and hA3G) Zn^++^-binding deaminase motifs with the consensus sequence His-X-Glu-X_23–28_-Pro-Cys-X_2–4_-Cys (where X denotes any amino acid) [63]. They can target cytosines and convert them to uracils (C to U transitions) on DNA or RNA (e.g. A1) templates. During the second-strand DNA synthesis, these C to U transitions are then converted to those of G to A. For example, by changing C6666 to U6666, A1 introduces a stop codon at position 6666 into the apolipoprotein B100 mRNA, which is translated into the truncated apolipoprotein B48 (48 kDa) protein [64]. AID also directs the cytidine deamination at specific "hot spots" to direct somatic hypermutation and isotype class switching in B cells [65]. hA3F and hA3G block retroviral infection in hematopoietic cells. They share overall 70% sequence similarity and form homodimers as well as mixed oligomers [5]. Physiological functions of these proteins are not yet defined, except that hA3G also inhibits the movement of some mouse mobile genetic elements in cells [66]. Thus, they could contribute to the stability of the genome.

## APOBEC proteins and viral replication

The mechanisms for antiviral activities of APOBEC proteins have been characterized extensively. In the absence of Vif, hA3F and hA3G are incorporated into virions. They are then transferred from producer to target cells by the virus. Following viral entry and uncoating, reverse transcription is initiated and viral minus-strand cDNA is synthesized. During this process, these APOBEC proteins attack newly synthesized minus-strand cDNAs and introduce C to U transitions [67-70], which block viral replication by several mechanisms [71]. First, since uracils are not tolerated in DNA, they are removed by uracil N-glycosidases (UNG) from DNA and these nicked DNA are further cleaved by the host DNA-repair enzymes like apurinic/apyrimidinic endonuclease-1 (APE1). Fragmented DNA neither integrates nor replicates. Second, should edited proviruses survive and integrate, the new G to A changes on the plus strand DNA also create havoc on viral transcripts. These changes could lead to alternate splicing and the production of nonfunctional proteins. To these ends, hA3G and hA3F have different sequence preferences. Whereas hA3G favors repeated deoxycytidines (GG on the opposite strand) [72], hA3F prefers deoxycytidines followed by deoxythymidines (GA on the opposite strand) [4,7].

The key step for the anti-viral activity of hA3G is its incorporation into virions. Although the encapsidation of hA3G requires NC of HIV-1 Gag, it is still controversial whether this interaction is mediated by RNA [73-76]. Since both NC and hA3G can bind RNA, this recruitment most likely reflects RNA-protein, as well as protein-protein, interactions [77,78]. Nevertheless, since hA3G blocks the replication of all primate lentiviruses in the absence of Vif (HIV-1, HIV-2, and SIV) [3,79,80], EIAV [69], HBV [81,82], and some mouse mobile genetic elements [66], these interactions must have broad specificities. For example, hA3F has the same effect against HIV-1, SIV and HBV and hA3B and hA3C block SIV (Table 1) [4-7,83,84]. Of interest, the rat but not human A1 proteins block HIV-1 by directly deaminating viral RNA [85].

**Table 1:** Abilities of APOBEC proteins to inhibit viruses and retrotransposons

Species	APOBEC proteins	HIV-1ΔVif	HIV-2ΔVif	SIVΔVif	MLV	EIAV	HBV	L1	IAP MusD
humans	A3B	+		+	-				
	A3C	-		+	-				
	A3F	+		+			+		
	A3G	+	+	+	+	+	+	-	+
old world monkeys	A3G	+		+	-				
rats	A1	+			-				
mice	A3	+		+	-			-	+

(+) block, (-) do not block

## Vif and APOBEC proteins

In contrast to HIV-1ΔVif, wild-type HIV-1 is not restricted in non-permissive cells. Thus, Vif counteracts the effects of hA3F and hA3G. Indeed, Vif binds and triggers the degradation of these APOBEC proteins in producer cells, thus blocking their incorporation into virions [86-88]. Initially, Vif was demonstrated to interact with cellular proteins Cul5, elonginB, elonginC, and Rbx1 to form a cullin-based E3 ubiquitin ligase complex [89], which displays striking similarities to SCF complex. Later, Vif was found to contain a conserved suppressor of cytokine signaling (SOCS) box-like motif (SLQ(Y/F)LA) that binds elongin C, which in turn recruits elongin B, cullin 5 and Rbx1, thus forming the ElonginB/C-Cul5-SOCS-box (ECS) E3 ubiquitin ligase complex [90,91]. As a consequence of these interactions, APOBEC proteins are polyubiquitylated and degraded [86-88]. In parallel, some groups observed that Vif triggers only a marginal degradation of hA3G, which suggested that Vif could sequester hA3G from encapsidation through a degradation-independent mechanism [79,92].

**Figure 5:**
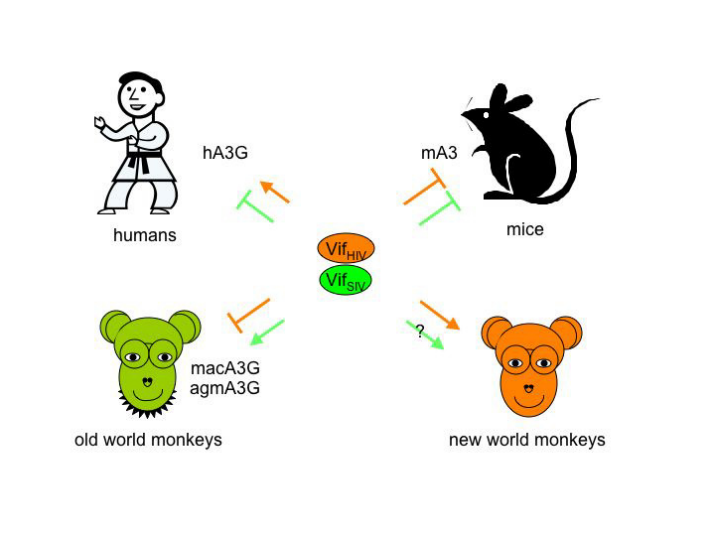
Species-specific inhibition of APOBEC3 (A3) proteins by Vif. HIV-1 and SIV can incorporate A3 proteins from all these species into virions. However, Vif from HIV-1 (Vif_HIV_, brown), can only inhibit A3 proteins from humans and new world monkeys) but not those from old world monkeys and mice. In contrast, Vif from SIV_mac _(Vif_SIV_, green) can inactivate A3 proteins from old and possibly new world monkeys, but not from humans and mice). In this drawing, stick figures represent sources of A3 proteins and not targets of infection.

Although Vif blocks the antiviral activity of hA3G, its activity is highly species-specific (fig. 5). Additionally as presented in Table 2, Vif from HIV-1 blocks A3G proteins from humans and chimpanzees (hA3G and chA3G), but not from old world monkeys, Vif from SIV_mac _blocks all A3G isoforms from human and non-human primates, and Vif from SIV_agm _only blocks A3G proteins from monkeys [79]. In addition, hA3F can be inactivated by Vif proteins from HIV-1, HIV-2, and SIV_mac_, but not from SIV_agm _[4,5,84]. Moreover, the hA3C can be inactivated by Vif from SIV_mac _[84] and no Vif protein can inactivate the hA3B, rat A1, or mouse APOBEC3 (mA3) proteins [6]. Efforts have been made to uncover the molecular mechanisms of these species-specific differences, and agmA3G was chosen because it is most similar to hA3G. It was found that Vif from HIV-1 fails to inactivate agmA3G because it contains a lysine rather than aspartate at position 128 (K128D), which is found in hA3G. Although agmA3G neither binds Vif nor is excluded from virions, the mutant agmA3G protein bearing D128 becomes fully sensitive to Vif from HIV-1 [93-96]. In addition, the reciprocal exchange of D for K at position 128 in hA3G renders it resistant to Vif. Structural comparisons with the related cytidine deaminases from *E.coli *reveal that D128 maps to an α-helical turn on an exposed loop [96]. Since the same K128 residue also exists in A3G from rhesus macaque (macA3G), its sensitivity might also be altered with a similar K128D substitution. Although these studies established the correlation between the ability of Vif to neutralize APOBEC proteins and viral replication, it is unlikely that these species-specific susceptibilities of APOBEC proteins to Vif are responsible for the transmission of primate lentiviruses to new host species [97].

**Table 2:** Species-specific susceptibility of APOBEC proteins to Vif

APOBEC proteins	Species	HIV-1 Vif	HIV-2 Vif	SIV_agm _Vif	SIV_mac _Vif
A3G	humans	+	+	-	+
	chimpanzees	+		-	+
	African green monkeys	-		+	+
	rhesus macaques	-		+	+
A3	mice	-		-	-
A3F	humans	+	+	-	+
A3B	humans	-		-	-
A3C	humans	-		+	+
A1	rats	-			

(+) susceptible, (-) not susceptible

## Viruses that lack Vif and mobile genetic elements

How can viruses that do not encode Vif escape from this intracellular immunity?

As some APOBEC proteins also inhibit the replication of EIAV, HBV, and MLV, there must be additional mechanisms of escape. In the case of EIAV, it encodes an additional enzyme, which is named dUTPase [98]. This enzyme is also produced by herpesviruses, poxviruses, and some other retroviruses. Recently, it was demonstrated that dUTPase from caprine arthritis encephalitis virus (CAEV) could block the misincorporation of dUTP during HIV-1 reverse transcription [99]. Thus, its dUTPase could also protect EIAV from attack by APOBEC proteins. In the case of HBV, it replicates in tissues that do not express hA3G [63]. The situation for MLV is more complicated. Unlike human cells that express seven A3 proteins, the mouse genome contains only one A3 gene. Although mA3 blocks the replication of HIV-1 and SIV, it is less efficiently packaged into and does not inhibit MLV [100]. In contrast, hA3G blocks the replication of MLV. Thus, MLV has adapted to its natural host by a mechanism that remains poorly understood.

What is the situation with mobile genetic elements that resemble retroviruses? In humans and mice, there are several types of retrotransposons [101]. The most abundant are LINE-1 or L1 elements that do not contain long terminal repeats (LTRs). Up to one hundred human and several thousand mouse L1 elements are functional [101]. Although they require reverse transcription, they do not form VLPs and APOBEC proteins do not block their replication [66,102]. In contrast, LTR-containing retrotransposons, which represent up to 10% of the human genome, form VLPs, bud from intracellular organelles and behave similarly to incoming exogenous retroviruses. Although no active human endogenous retroviruses (HERVs) have been found, in the mouse, there are several hundred active intracisternal A-particles (IAPs) [103] and at least ten copies of MusD [104]. Indeed, sequences of IAPs and MusDs contain frequent G to A transversions in their genomes [66]. In addition, using transient expression assays in cells, hA3G and mA3 inhibit the retrotransposition of IAP and MusD (Table 1) [66]. Thus, APOBEC proteins also block the movement of some mobile genetic elements, most likely in germ cells and during embryogenesis, in mammals.

## Other antiviral genes that contribute to intracellular immunity

Besides these predominant blocks to viral replication in cells, additional barriers have been described at levels of transcription and RNA stability, as well as assembly of progeny virions. However, since they do not block the integration of proviruses into the host genome, they play lesser roles in intracellular immunity. First, Murr1 blocks the activation of NF-κB in resting cells and thus the induction of HIV-1 replication [105]. Second, a sophisticated genetic screen looking for cells that survive attack by MuLV bearing the thymidine kinase (tk) gene (which would otherwise succumb to trifluorothymidine that is phosphorylated by tk) revealed the Zn^++^-finger antiviral protein ZAP that degrades rapidly MLV transcripts [106]. ZAP binds a specific sequence at the 3' end of viral, but not cellular, transcripts and leads to their rapid degradation in the exosome. This mechanism appears analogous to tristetraprolin, which binds AU-rich RNA species (e.g. those coding for cytokine genes) and targets them for rapid degradation in the cytoplasm. Apparently, not only are retroviral transcripts targeted by ZAP, but it destroys Ross River, Semliki, Sindbis and Venezualan equine encephalitis viruses, all of which belong to the alphavirus family [107]. Alghough ZAP is extremely efficient againt alphaviruses and MuLV, it is not clear what role, if any, it plays against primate retroviruses.

The final level of intracellular immunity deals with viral assembly and release. Again, HIV-1 encodes another accessory viral protein u (Vpu), which facilitates the release of progeny virions from infected cells [108]. Thus, analogous to the situation with Vif, some cells are "permissive" and others are "non-permissive" for viral replication in the absence of Vpu. Heterokaryons between them maintain the non-permissive phenotype, which is dominant. Thus, Vpu must counteract some dominant negative cellular factor, whose identity remains to be determined. Of interest, recent work suggests that Vpu counteracts the two-pore K+ (K_2P_) channel TASK-1, which inhibits the release of many viruses by an unknown mechanism, possibly by changing membrane fluidity [109]. Vpu also facilitates the release of other retroviruses. By mimicking a natural component of TASK-1, Vpu is incorporated into the channel, where it acts as a dominant negative effector. Vpu also binds βTRCP, an E3 ubiquitin ligase, which could accelerate the degradation of TASK-1 in the proteasome [110]. Thus, it is possible that levels and/or polymorphisms of TASK-1 are mostly responsible for this block in the assembly and release of progeny virions. However, additional experiments are required to make this connection and/or to reveal additional players in this last step of the viral replicative cycle in cells.

## Intracellular immunity

Several themes emerge from these cell-intrinsic blocks to retroviral replication. First, the inhibition is broad. Thus, not only are retroviruses targeted, but other viruses as well, from HBV and alphaviruses to some mobile genetic elements, which once were viruses themselves. Second, multiple steps in the replicative cycles of these viruses are inhibited, most likely because each mechanism is not completely effective. This finding might reflect small differences between extracellular pathogens and normal cellular homeostatic mechanisms. Alternatively, it might reflect the vast spectrum of different pathogens, all of which must be targeted and destroyed. For retroviruses, the challenge is increased because of their rapid rate of mutations and their quick adaptation to the host. Third, these intracellular blocks are more pronounced and effective in zoonotic infections, where the virus jumps species. Finally, this inhibition is rapid and targets predominantly early steps in the replicative cycles of these viruses. Thus, it tries to prevent the integration of the viral genetic material into the host genome. Whether these inhibitors accomplish this task by targeting viral structures or genetic material to an endosome, exosome or proteasome, the end results are the same, i.e. the elimination of the virus. In this scenario, the outcome depends on the effectiveness of cellular proteins versus the protective armor of the virus.

Given these observations, one of the simplest new therapeutic interventions could be simply to increase intracellular levels of these antiviral proteins, e.g. TRIM5α, APOBEC proteins, ZAP and/or TASK-1. Thus, if we only understood their normal regulation, it is possible that we could augment their amounts and activities during active infections. Of course, as we do not know their other functions in cells, there are also many potential concerns. For example, would increased levels of APOBEC3G cause editing of genomic DNA during replication, thus facilitating oncogenic transformation? Likewise, would increased amounts of ZAP target critical cellular transcripts for accelerated degradation? Alternatively, one could try to block interactions between Vif and APOBEC proteins and TASK-1 and Vpu. Possibly, by studying their structures, one could design inhibitors for their protein-protein interactions. Moreover, all these processes can also be targeted by gene therapy, by introducing into cells their counterparts from different species and/or by changing binding surfaces of the host proteins so that they no longer interact with Vif or Vpu, for example. If not practical clinically, such genetic manipulation would yield important clues as to which restriction should be targeted by other therapeutic means.

## Conclusion

It is remarkable how active are the processes that protect an organism from internal and external challenges. In humans, at least three layers of immunity have developed. Among them, intracellular and innate immune responses act primarily via pattern recognition, whereas adaptive immunity is very sequence and peptide-specific. Nevertheless, many pathogens break through and are integrated into the host genetic material. Thus, some of these defense mechanism must also survey the movements and effects of these mobile genetic elements. It appears that a fine line has been drawn between control and allowing for some escape as well. As mobile genetic elements contribute to evolution and fitness of all species, they must be kept in check, but not eliminated completely and it is possible that this intricate regulation of hA3G activity in cells reflects this requirement. On the other hand, there is also a high price to pay in terms of mistakes, be they developmental defects or cancer. Nevertheless, the study of these systems that fight extracellular pathogens is likely to reveal fundamental insights into a plethora of cellular processes that contribute to human health and disease.
